# Supplementary material for: Splice-Junction-Based Mapping of Alternative Isoforms in the Human Proteome
Source: Cell Rep. Author manuscript; Available in PMC 2020 Jan 15. (PMC6961840; doi:10.1016/j.celrep.2019.11.026)
Supplement: 2 [file NIHMS1546469-supplement-2.zip › DF1/PXD010154/Heart/Heart_77_DMD_EEDFNKDMDLR.pdf]

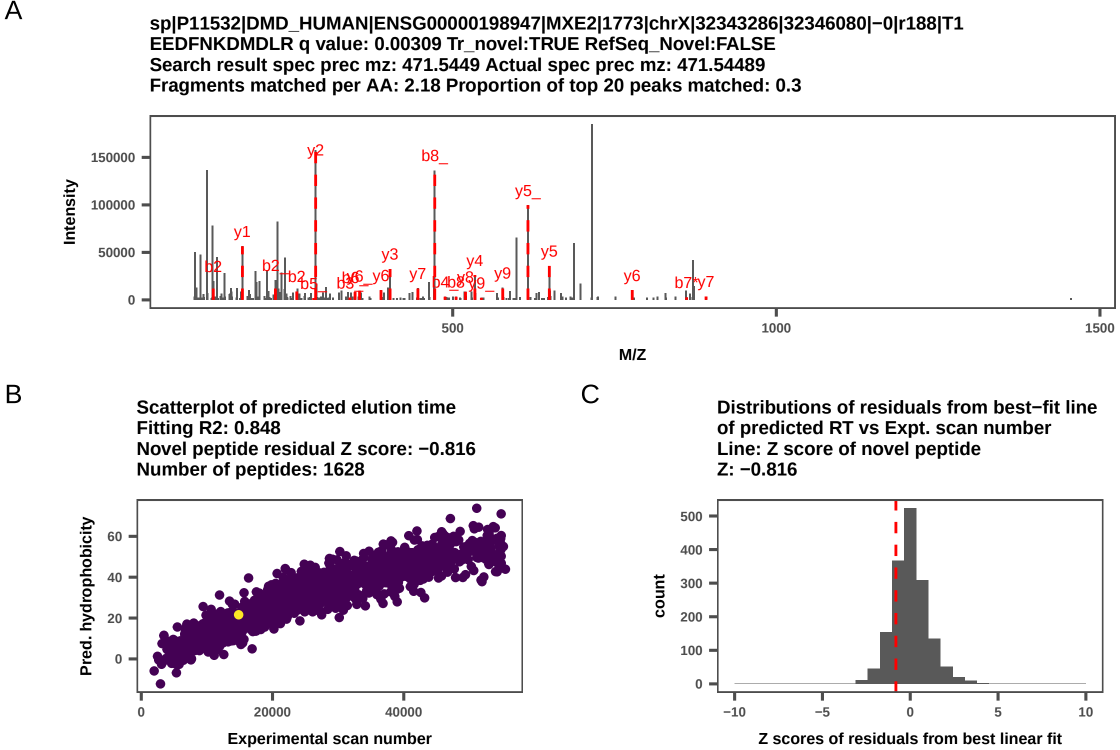
A
sp|P11532|DMD_HUMAN|ENSG00000198947|MXE2|1773|chrX|32343286|32346080|−0|r188|T1
EEDFNKDMDLR q value: 0.00309 Tr_novel:TRUE RefSeq_Novel:FALSE
Search result spec prec mz: 471.5449 Actual spec prec mz: 471.54489
Fragments matched per AA: 2.18 Proportion of top 20 peaks matched: 0.3
Intensity
150000
100000
50000
0
M/Z
500
1000
1500
y2
b8_
y5_
y1
y5
y3
y4
y9
y6
y7
B
Scatterplot of predicted elution time
Fitting R2: 0.848
Novel peptide residual Z score: −0.816
Number of peptides: 1628
Pred. hydrophobicity
60
40
20
0
0
20000
40000
Experimental scan number
C
Distributions of residuals from best−fit line
of predicted RT vs Expt. scan number
Line: Z score of novel peptide
Z: −0.816
count
500
400
300
200
100
0
−10
−5
0
5
10
Z scores of residuals from best linear fit
